# Comparing the intraoperative and postoperative complications of the scalpel and electrocautery techniques for severing the inner layers of the lumbar disc during discectomy surgery

**DOI:** 10.3389/fsurg.2023.1264519

**Published:** 2023-09-28

**Authors:** Parisa Hajilo, Behzad Imani, Shirdel Zandi, Ali Mehrafshan

**Affiliations:** ^1^ Student Operating Room, Student Research Committee, Hamadan University of Medical Sciences, Hamadan, Iran; ^2^ Department of Operating Room, School of Paramedicine, Hamadan University of Medical Sciences, Hamadan, Iran; ^3^ Department of Operating Room, School of Paramedicine, Hamadan University of Medical Sciences, Hamadan, Iran; ^4^ Department of Neurosurgery, Nekuii Forghani Hospital University of Medical Sciense Qom, Qom, Iran

**Keywords:** scalpel, diskectomy, incision, stainless steel scalpel, electrocauter

## Abstract

**Background:**

Due to the sensitivity of the surgical site and a higher probability of injury, the use of a scalpel and electrocautery to create an incision in the spine is discussed. In this study, we will compare the intraoperative and postoperative complications of the scalpel and electrocautery techniques for severing the inner layers of the lumbar disc during discectomy surgery.

**Materials and methods:**

This study was conducted in Iran as a randomized controlled trial with double-blinding (1,401). Sixty candidates for spine surgery were randomly divided into two groups of 30 using electrocautery (A) and a scalpel (B) based on available sampling. The VAS scale was used to assess postoperative pain. The duration of the incision and intraoperative blood loss were recorded. The infection and fluid secretions were determined using the Southampton scoring scale. Utilizing the Manchester scar scale, the wound healing status was evaluated. The SPSS version 16 software was used for data analysis (*t*-test, Mann–Whitney *U*, ANOVA).

**Results:**

The electrocautery group had substantially lower bleeding, pain, and wound healing rates than the scalpel group (*P* > 0.05). However, the electrocautery group had significantly longer surgical times, more secretions, and a higher infection rate than the scalpel group (*P* > 0.05). In terms of demographic and clinical characteristics, there was no significant difference between the two groups (*P* < 0.05).

**Conclusion:**

Electrocautery reduces postoperative hemorrhage and, potentially, postoperative pain in patients. However, as the duration of surgery increases, so does the duration of anesthesia, and patient safety decreases. Additionally, the risk of infection increases in the electrocautery group compared to the scalpel group, and the rate of wound healing decreases.

**Clinical Trial Registration:**

https://www.irct.ir/, identifier (IRCT20230222057496N1).

## 1. Introduction

Controlling bleeding during surgery is the primary concern of all surgeons, and its significance increases in significant procedures, such as head-and-neck and spine operations (1). Lumbar discectomy is one of the most common surgical proceduresa for treating spine disorders in patients who do not respond to conservative treatment (2). Due to their adverse position and excessive blood loss, these patients require special care during surgery (3). On the other hand, vascular hemostasis during surgery lengthens the duration of the procedure and jeopardizes patient safety (4). With the advancement of technology, electrocautery entered the field of surgery as a widely used device in the 20th century, attracting the attention of surgeons (5) due to its high hemostasis power during cutting. Electrocautery transmits electric impulses from cell to cell to the desired tissue and, by producing heat, causes tissue severing and vascular coagulation (6). Scalpels are also extensively used as a traditional method of surgical cutting due to their precision and minimal tissue damage (7). However, cutting with a scalpel has several drawbacks that limit its application. One of the most significant disadvantages is hemorrhaging, a primary concern for anesthesia and surgical teams (6). In addition, transmitting viruses such as AIDS, hepatitis B, and hepatitis C through scalpel wounds is widespread (8). However, electrocautery with ligation of the surgical site's blood vessels reduces the likelihood of malignancy metastasis through lymphatic pathways and contamination of the surgical team with viral infections (9). Consequently, the form of surgical incision is a subject of discussion during surgical procedures (10). In addition to the quantity of bleeding, the type of incision in surgery affects factors such as the duration of surgery, infection, wound healing, and patient pain, according to the findings of several studies (6, 11). The pain following surgery has substantially impaired the patient's quality of life, mobility, and performance (12). Katsuda et al. (13) theorize that nerve terminals are heated by electric current in electrosurgical incisions of spine surgeries, and patients experience less discomfort during the recovery phase (13). A study found that using electrocautery for tissue dissection increases postoperative pain, necrosis, and severe tissue injury (14).

Today, some surgeons, immediately after cutting the skin with a scalpel, create incisions in all the layers beneath the skin, fascia, and muscle using electrocautery to minimize the disadvantages of the scalpel (10). Some surgeons hesitate to use electrocautery due to its undesirable disadvantages (15), despite its numerous benefits. One of its main disadvantages is burns and tissue necrosis caused by the heat of electrocautery, which spreads to nearby tissues and causes a delay in wound healing (16). Additionally, inhaling cautery smoke causes irritation of the eyes and pharynx, as well as respiratory, digestive, and cardiovascular disorders (17). In their study, Shah Akbari et al. (18) found that electrocautery-induced thermal injury to adjacent tissues results in the formation of hypertrophic tissue in the surgical area (18). However, in the study by Zarei et al. (19), there was no statistically significant difference in wound healing speed and patient satisfaction with cosmetic results (19). Furthermore, according to Nagargoje et al. (10), using electrocautery in tissue cutting increases the surgical time and negatively affects patient wound healing (10). Despite this, the results of numerous other studies indicate that the electrocautery group cuts at a substantially slower rate than the scalpel group (16, 20).

The contradictory results of studies conducted in Iran and other countries regarding the advantages and benefits of electrocautery and scalpel indicate that people do not have sufficient knowledge in this area, despite the widespread use of these two instruments (21). Consequently, the purpose of this study is to compare the side effects during and after surgery of the techniques of using a scalpel and electrocautery in cutting the inner layers of lumbar discectomy surgery so that, by providing more detailed information in this field, a consensus can be reached regarding the benefits and drawbacks of these two widely used instruments.

## 2. Methods

### 2.1. Study design

This study is a randomized, double-blind clinical trial (IRCT20230222057496N1) conducted in Iran Teaching Hospital (Qom Province) in 1,401. All patients underwent surgery under general anesthesia. The electrocautery voltage was set to the same level for all patients (70 J monopolar, 100 J bipolar) to assess the bleeding quantity accurately. In both groups, the bipolar thermal flow was used to occlude bleeding vessels. The fascia and subcutaneous tissues were repaired with two vicryl and one vicryl thread, respectively, and the skin was sutured with 2.0 nylon thread. All patients were matched from the beginning of anesthesia to the administration of analgesics in the recovery department to determine the effects of anesthetic drugs on the risk of infection or postoperative pain in patients. Fentanyl was used for both the induction and maintenance of anesthesia, measured in 2 µg and 2 µg/kg respectively. During surgery, 3 mg of morphine was administered intravenously. Patients were injected with cefazolin at the outset of 2 g surgery to control infection. In addition, patients in the recovery department were injected with 1 mg/kg of pethidine to alleviate their discomfort. Other medications administered in the hospital and after discharge were identical.

### 2.2. Setting and participants

Patients between the ages of 30 and 50 with intervertebral disc herniation (one-lobe disc herniation in the range of L1-S1) eligible to participate in the study. Patients who are reticent to be followed up after surgery or who reside outside the province are also excluded from the study.

### 2.3. Sample size calculation

The aim of this study was to compare the mean values of quantitative variables in two electrocautery (group A) and scalpel (group B) groups. The required sample size to achieve the desired analytical objectives was calculated using the following formula, which has been introduced in various sources as an appropriate method for determining sample size for hypothesis testing of the difference between the means of two populations (22, 23). Based on the introduced formula and the mean scores obtained from Shahakbari et al. (2017) study, which reported these values as 1.4 ± 0.33 and 1.73 ± 0.47 for the scalpel and electrocautery groups, respectively (24), and considering a confidence level of 95% and a test power of 80%, the sample size in each group was calculated to be 24 individuals. Taking into account a 20% potential dropout rate, the final sample size in each group was determined as 30 individuals.

$$n=\frac{{\left(z_{\alpha }+z_{\beta }\right)}^{2}(s_{1}^{2}+s_{2}^{2})}{{\left(\mu_{1}-\mu_{2}\right)}^{2}}$$

### 2.4. Randomization and allocation

Based on the permuted block design, patients were randomly divided into two electrocautery (group A) and scalpel (group B) groups. Blocking was used to distribute equal samples to each study group. Electrocautery and scalpel (blade 20) were used to cut the internal layers of the lumbar surgery area for patients in the two study groups. The allocation was concealed using a sealed envelope that was unsealed in the operating room after the patient's induction. Patients and the main researcher were blinded to the surgical procedure and group.

### 2.5. Scalpel group

For patients who required lumbar dissection as part of the treatment protocol, this incision (blade 20) was made with a scalpel.

### 2.6. Cautery group

Patients required dissection of the lumbar region as part of the treatment protocol, which was performed using electrocautery.

### 2.7. Validity and confounding factors

Excluded from the study were patients with a history of lumbar surgery, anemia, rheumatoid arthritis, osteoporosis, hypertension, uncontrolled diabetes, any active infection in the body, and those taking anticoagulants or any medication that affects wound healing. Some of the confounding factors of the study, including the stressful personality type of people, which can affect the level of pain after surgery and wound healing. Psychological problems of patients that can be an obstacle to improve pain after surgery, which were controlled by personality tests and pre-surgery screening form in the study. Due to the unfavorable position of the patients, the surgery in the lumbar region reduces the urinary output and increases the amount of bleeding during the surgery due to the pressure on the inferior vena cava, which was controlled by using DVT pumps during the surgery.

### 2.8. Instruments and measures

In addition to a thorough history and physical examination, sociodemographic and clinical data were collected from the patients. One of the surgeon's assistants kept track of the duration of the incision and the quantity of blood loss during the operation. To determine the quantity of bleeding during surgery, 10 × 10 gas swabs were weighed (with a precision of 1 gr) and were used exclusively for creating incisions and during hemostasis. Additionally, the blood content in the suction bottle was calculated regarding CC. Before surgery, the difference in weight between dry and moist gases was considered.

#### 2.8.1. VAS scale

Visual Analogue Scale (VAS) was used to record patients' pain levels from the time they entered the department to the first, seventh, and fourteenth days following surgery. The Pain Scale is identical to the 0 to 10 pain rating scale. Zero indicates a complete absence of pain, while ten indicates unbearable pain. The person's position on the continuum is determined by their pain intensity over the previous 48 h.

#### 2.8.2. Southampton

Using the Southampton wound Scoring System, the wound was evaluated clinically every postoperative day for surgical site infection up to one month after surgery. On this scale, a surgical site infection with erythema was considered grade 1. Grade 2 findings are identical to grade 1 findings with serous fluid. Grade 3 is identical to grade 2 with cloudy infectious fluid in half the incision, and grade 4 is identical to grade 3 in more than half the wound (25).

#### 2.8.3. Manchester score scale

In addition, the wound healing status of patients in both groups was assessed using the Manchester Scar Scale (MSS) on the 7th and 30th day after surgery (evaluation of color, contour, wound surface curvature, and keloid tissue). The total score obtained was (4–14) with a score of 4 indicating excellent wound healing and a score of 14 indicating poor wound healing (26).

### 2.9. Data collection and statistical analysis

The SPSS version 16 software was used for data analysis. Using the Shapiro-Wilk Test, the data's normality was determined. Independent-Samples The T-test was used to compare the variables of body mass index, hemorrhage rate, and surgical time between the A and B groups. Mann-Whitney The U test compared the wound healing, infection, and fluid secretion rates between groups A and B. The significance of the effect of time and group on the pain variable was determined using a one-way repeated-measures ANOVA test. Also, from this test, the significance of the simultaneous effect of time and gender on the amount of pain in groups A and B was investigated separately in order to determine its significance. Every test was conducted with a margin of error of 0.05.

### 2.10. Ethical considerations

Hamedan University of Medical Sciences Ethics Committee approved the study (IR.UMSHA.REC.1401.1005). The study's nature, methodology, and risks were explained to the patients, and all patients provided written informed consent. In addition, the Declaration of Helsinki principles were observed in this study.

## 3. Results

The results of the Shapiro-Wilk test indicate that, at the 95% confidence level, the variables of body mass index, hemorrhage rate, and surgery time in both groups A and B follow a normal distribution (*P* > 0.05).

### 3.1. Comparison of variables related to body mass index, hemorrhage rate, and surgical time for groups A and B

According to the parametric independent *t*-test results, the mean variable of body mass index for groups A and B does not differ significantly (*P* > 0.05). However, there is a significant difference between the variable means of hemorrhage rate for groups A and B. Consequently, the quantity of bleeding in group B (scalpel) is significantly greater than in group A (electrocuting) (*P* < 0.05). Also, there is a statistically significant difference between the two groups, A and B, about the mean variable of surgery duration. Group A (electrocautery) has a substantially longer surgical time than Group B (scalpel) (*P* < 0.05) (Table 1).

**Table 1:** The results of Levene's test and independent *t*-test to compare the variables of body mass index, bleeding rate and surgery time according to two groups A and B.

Variable	The results of the homogeneity of variances test (Leven)		The results of the equality of means test (independent *t*)	
	Sig	*F*	Sig	*T*
BMI	0.052	3.924	0.182	−1.349
Blood loss	0.720	0.130	0.000	−9.123
Surgical time	0.124	2.440	0.000	5.133

### 3.2. Variance analysis of repeated one-way measurements (significant study of the effect of time and group on the variable of pain level)

In Table 2, descriptive statistics for the pain level variable at any time are reported separately for groups A and B.

**Table 2:** Descriptive statistics of pain level according to two groups A and B.

Variable	Time	Group	Number	Mean	Standard deviation
Pain level	First day	A	30	9.13	0.819
		B	30	9.37	0.615
		Total	60	9.25	0.728
	7th day	A	30	6.97	1.245
		B	30	8.23	1.040
		Total	60	7.60	1.304
	14th day	A	30	3.63	1.066
		B	30	6.17	1.085
		Total	60	4.90	1.664

The results of the Box's *M*-test show that the hypothesis of the equality of the variance matrices of the dependent variable is accepted among different groups (*P* > 0.05). Also, Moschel's test of sphericity examines the congruence of the error covariance matrix related to the dependent variable normalized to an identity matrix. Because the value of the significance level of this test is equal to 0.037 and this value is smaller than the error level of 0.05, the assumption of sphericity of the variance-covariance matrix of the dependent variable cannot be accepted, and the Greenhouse-Geisser test was used in the interpretation of the table of the within-subjects effects tests.

The results of Table 3 show that the effect of time on the dependent variable of pain level is significant and this means that there is a significant difference between the average pain level on different days. So that with the passage of time, the average amount of pain decreases. Also, the interactive effect of time and group variables is significant, and these two variables have an interactive and simultaneous effect on the average pain level.

**Table 3:** The results of the Greenhouse-Geisser test to investigate the effects within-subjects.

Source	Df	Mean square	*F*	Sum of squares	Sig
Time	1.804	320.8	301.05	578.7	0.000
Time*group	1.804	22.07	20.71	39.8	0.000

The test results of effects between subjects are displayed in Table 4. As can be seen, the group effect on the dependent variable of pain level is significant, indicating that the average pain level in groups A and B is distinct. Therefore, the typical pain level in group B (scalpel) is more significant than in group A (electrocuting).

**Table 4:** The results of the Greenhouse-Geisser test to investigation the effects between subjects.

Source	Df	Mean square	*F*	Sum of squares	Sig
Group	1	81.339	75.590	81.339	0.000

### 3.3. Comparison of infection variable and serous fluid between the A and B groups

The results of the non-parametric Mann-Whitney test indicate that the levels of infection and serous fluid in groups A and B differ significantly. So the incidence of infection and serous fluid is markedly higher in group A (electrocuting) than in group B (scalpel) (*P* < 0.05) (Tables 5, 6).

**Table 5:** The mean rank of variable infection and serous fluid according to two groups A and B.

Variable	Group	Number	Mean ratings
Infection and serous fluid	A	30	33.50
	B	30	27.50

**Table 6:** The results of the Mann–Whitney test to compare the variable of infection and serous fluid according to two groups A and B.

Variable	*Z*	Sig
Infection and serous fluid	−2.260	0.024

### 3.4. Significant investigation of the effect of time and group on the variables of wound healing on the 7th day and the 30th day

The nonparametric Mann-Whitney test results indicate a significant difference between the wound healing rates of groups A and B on days 7 and 30. Thus, the rate of wound healing on the seventh and thirty-first day is substantially higher in group B (scalpel) than in group A (electrocautery) (*P* > 0.05) (Tables 7, 8).

**Table 7:** Average ranking of wound healing variables on the 7th and 30th days according to two groups A and B.

Variable	Group	Number	Mean ratings
Wound healing on the 7th day	A	30	20.60
	B	30	40.40
Wound healing on the 30th day	A	30	20.50
	B	30	40.50

**Table 8:** Results of the Mann–Whitney test to compare wound healing variables on the 7th and 30th days according to two groups A and B.

Variable	*Z*	Sig
Wound healing on the 7th day	−4.878	0.000
Wound healing on the 30th day	−5.121	0.000

### 3.5. Gender-based comparison of variables about body mass index, the amount of bleeding, and length of surgery in groups A and B

The results of the parametric independent *T*-test indicate that the average variable of body mass index does not differ significantly between the two categories of men and women in groups A (*P* > 0.05) and B (*P* > 0.05). In both male and female groups, the variable mean of the hemorrhage rate for groups A (*P* > 0.05) and B (*P* > 0.05) was reported to be the same. In addition, the average variable of surgery time for group A (*P* > 0.05) and group B (*P* > 0.05) was not statistically significant (Table 9).

**Table 9:** The results of Levene's test and independent *t*-test to compare the variables of body mass index, bleeding rate and surgery time in groups A and B according to gender.

Variable	Group	The results of the equality of means test (independent *t*)		The results of the homogeneity (Leven) of variances test	
		Sig	*t*	Sig	*F*
BMI	A	0.979	0.027	0.302	1.104
Blood loss		0.299	1.058	0.228	1.518
Surgical time		0.225	1.241	0.649	0.212
BMI	B	0.865	0.171	0.012	7.277
Blood loss		0.430	−0.800	0.970	0.001
Surgical Time		0.350	0.950	0.496	0.475

### 3.6. Comparison of gender-based infection variable and serous fluid groups A and B

The results of the nonparametric Mann-Whitney test indicate that the infection rate and serous fluid of groups A and B in the two groups of men and women do not differ significantly (*P* > 0.05) (Table 10).

**Table 10:** Results of the Mann–Whitney test to compare the variable of infection and serous fluid of groups A and B according to gender.

Variable	Group	*Z*	Sig
Infection and serous fluid	A	−0.624	0.533
	B	−1	0.317

## 4. Discussion

So far, researchers have performed epidermal and mucosal incisions on human and animal samples using a scalpel and electrocautery (25, 27). Despite a comprehensive search of databases, we could not find a study that directly compared scalpel and electrocautery in lumbar surgery. The purpose of the present study was to compare the intraoperative and postoperative complications of the scalpel and electrocautery techniques for cutting the inner layers of the lumbar discectomy. The results indicate that the electrocautery group experienced statistically less hemorrhage than the scalpel group. In this regard, Sheikh's (7) study on 177 patients undergoing brain surgery revealed that electrocautery results in substantially less bleeding than a scalpel (7). In addition, Kumar et al. (28) found the following in a study of 80 patients undergoing head and neck surgery. Due to the importance of controlling bleeding in head and neck surgery, the surgical site must be coagulated entirely before the operation can commence. Electrocautery is regarded as a safer instrument than the scalpel (28). Marsh et al. (29) obtained comparable outcomes with abdominoplasty (29). Electrocautery appears to increase hemostasis by closing blood vessels before cutting. In other words, electrocautery utilizes thermal energy to denature proteins, and this alteration in protein conformation results in vascular tamponade and tissue homeostasis.

The results of the study indicate that patients in the electrocautery group experience substantially less pain than those in the scalpel group. The results of a meta-analysis of comparative studies in this field were reported to substantiate the findings above (6). In hernia surgery, Ragesh et al. (25) demonstrated that the electrocautery group experienced substantially less postoperative pain than the scalpel group (25). The study by Shamim et al. (30) also indicates that the electrocautery group experienced less discomfort after surgery and required only half the amount of painkillers as the scalpel group (30). Several additional studies found similar outcomes (31, 32). Alizadeh et al. (1) contend that electrocautery-assisted skin incision increases patient pain, burns, and tissue necrosis after surgery (1). Due to thermal damage to the nerve endings in the tissue layers, electrocautery-performed tissue dissection of internal layers substantially reduces postoperative pain in patients. Due to the accumulation of peripheral nerves around the facet joints and paravertebral muscles, this reduction in discomfort during spine surgery is more apparent. As a result of extensive thermal damage to the skin's surface, it causes skin burns, pain, and burning in the surgical area when applied to skin incisions.

The data analysis indicates that the electrocautery group's surgical cutting time is substantially longer than the scalpel group's. The results of most studies contradict those of the present study (20, 31, 32). Shah Akbari et al. (18) findings in orthognathic surgeries indicate that using a cauter reduces surgical time compared to using a scalpel (18). In this regard, Prakash et al. (9) reported that the average surgical cutting time between the electrocautery and scalpel groups was not substantially different (9). Numerous studies may have yielded inconsistent results due to the sensitivity of the targeted area for cutting and surgery. In the initial investigation, lumbar surgery is performed. Due to the spinal cord's and nerve roots' vicinity during electrosurgical dissection, the surgeon must proceed with greater sensitivity and precision to avoid damaging vital tissues. This resulted in a more extended surgical procedure for the electrosurgery group than other investigations. The results of a study conducted in the neck region by Thakare et al. (33) indicate that using electrocautery increases the surgical time in the spine region (33).

Infection and serous fluid secretions are substantially less prevalent in the scalpel group compared to the electrocautery group. In gynecological interventions, Franchi et al. (34) found that the risk of infection following electrocautery tissue cutting and dissection is greater than that following scalpel tissue cutting and dissection (34). In contrast to the findings above, Ragesh et al. (25) found no statistically significant difference between the electrocautery and scalpel groups regarding infection incidence and serous fluid secretions (25). Contradictory results of studies due to infection in electrocautery cutting may be caused by the degree of sterility of the surgeries, how patients care for their wounds at home, and numerous other factors considered study limitations and cannot be controlled or prevented.

In the electrocautery group, wound healing and the formation of colloidal scar tissue are statistically lower than in the scalpel group. In thyroidectomy surgery, Uludag et al. (4) reported that the heat applied to the adjacent tissue during electrosurgery caused nerve injury in the area, slowed wound healing, and produced unsatisfactory aesthetic results (4). Also, in the Shah Akbari et al. (18) study, scar tissue was more significant in the electrocautery group than in the scalpel group (18). Zarei et al. (19), with a 3-month follow-up after abdominal hernia surgery, reported no significant difference between the two groups regarding wound healing speed and scar tissue (19). Ismail et al. (6) reported comparable findings from a meta-analysis of 41 studies (6). Depending on the patient's skin type, the type of suture used in the incision area, the depth of the dissected tissue, the level of patient satisfaction with wound appearance, the formation of keloid scar tissue, and wound healing varies. In addition, the type of dissected tissue will influence wound healing differently depending on the quantity of blood supply and anatomical condition.

In general, patients who undergo surgery with electrocautery are more satisfied and experience a shorter recovery period. The ability to adjust the input current in the electrocautery, unlike the scalpel, provides the conditions for the surgeon to proceed with more precision and concentration, preventing the possibility of damage to the patients' vital tissues, and increasing the safety of the patients. Also, by controlling the incoming flow, the possibility of injury to the surgical team and needle stick is potentially controlled and the safety of the employees is maintained. On the other hand, the selection of new surgical methods increases the mental and spiritual preparation of patients and has a potential impact on the outcome of surgery and the recovery of patients. But despite the many advantages of electrocautery, this tool cannot completely replace scalpel because the choice of surgical method depends on the preference and comfort of the surgeon and their experience. Our study has limitations, however. Only patients who met the study entry criteria were included using the available sampling procedure. In this regard, conducting studies with more precise sampling methodologies is necessary. In addition, the limited number of samples in our study is another limitation, and it is suggested that similar studies with a more significant number of samples be conducted at other medical centers.

## 5. Conclusion

Electrocautery reduces blood loss and improves the surgeon's line of vision. Additionally, electrocautery may reduce postoperative patient discomfort. Due to the sensitivity of the surgical area, however, the anesthesia time increases, and patient safety decreases as the duration of surgery increases. Therefore, more significant consideration must be given to the position of the surgical area when selecting a cutting instrument. Although the electrocautery group has a higher risk of wound infection and keloid scar formation, the risk of wound infection and keloid scar formation is decreased. However, the results of the currently available evidence are consistent with the substantiation of electrocautery use. This research was conducted on patients with no underlying disease; other confounding variables were omitted. It is recommended that, in future studies, participants with background issues affecting existing results be included to obtain more comprehensive results in this field.

## Data Availability

The original contributions presented in the study are included in the article/Supplementary Material, further inquiries can be directed to the corresponding author.
